# Ma’s bamboo-based medicinal moxibustion therapy of low back pain in lumbar disc herniation: study protocol for a randomized controlled trial

**DOI:** 10.1186/s13063-022-06382-x

**Published:** 2022-05-28

**Authors:** Xian-zhu Wang, Kai-yang Xue, Ping-nan Chen, Cai-hong Xiao, Jin Cui, Jing Fu

**Affiliations:** 1 Guizhou University of Traditional Chinese Medicine, Guiyang, 550002 China; 2 First Affiliated Hospital of Guizhou University of Traditional Chinese Medicine, Guiyang, 550001 China

**Keywords:** LDH, Low back pain, Ma’s bamboo-based medicinal moxibustion therapy, Acupuncture, Randomized controlled trial

## Abstract

**Background:**

Lumbar disc herniation (LDH) is a common and frequently occurring disease in clinics. Low back pain and sciatica are the presenting symptoms of LDH. To some extent, it can be considered that measures with the capability to improve low back pain or sciatica have the potential to treat LDH. Ma’s bamboo-based medicinal moxibustion therapy can effectively reduce the degree of low back pain and has been widely used. Studies of small sample size have seen significant improvement on pain relief. The aim of this trial is to evaluate the clinical efficacy and safety of Ma’s bamboo-based medicinal moxibustion therapy in the treatment of LDH low back pain.

**Methods/design:**

The trial is a multicenter, randomized, parallel-group, non-inferiority study. Three hundred and twelve patients will be randomly assigned to a Ma’s bamboo-based medicinal moxibustion group (*n*=156) and an acupuncture group (*n*=156). Patients in each group will receive treatment every day, 6 times a week, 12 times in total. Follow-up will be conducted 14 days after treatment. The primary outcome will be the visual analog scale(VAS) at baseline, after 6 times of treatment, end of treatment, and follow-up. The secondary outcomes will include Oswestry disability indexes (ODI), modified Japanese Orthopaedic Association low back pain (M-JOA) score, serum β-endorphin (β-EP), and serum substance P (SP). β-EP and SP, as well as safety evaluation indexes (routine blood, liver, and kidney function and electrocardiogram), will be measure at baseline and after the end of treatment. The number, nature, and severity of adverse events will be recorded.

**Discussion:**

The results of the trial will compare the efficacy of low back pain in LDH between Ma’s bamboo-based medicinal moxibustion group and the acupuncture group and will be expected to make a systematic and objective evaluation of the clinical efficacy and safety of Ma’s bamboo-based medicinal moxibustion therapy.

**Trial registration:**

ChiCTR2000038725. Registered on 29 September 2020.

**Supplementary Information:**

The online version contains supplementary material available at 10.1186/s13063-022-06382-x.

## Background

Lumbar disc herniation (LDH) is a syndrome caused by the compression and stimulation of the lumbosacral nerve root and cauda equina nerve owing to the degeneration of the lumbar intervertebral disc, rupture of annulus fibrosus, and resulted nucleus pulposus protrusion. It occurs frequently in young and middle-aged individuals, with a higher incidence in males than females, among which L4/5 and L5/S1 are the vulnerable sites (>90%) [1]. Low back pain is the initial symptom of LDH, which is generally in the lumbosacral part, mostly soreness and swelling pain, which can radiate to the buttocks. It occurs repeatedly, aggravates after sitting, standing for a long time, or fatigue, and relieves after rest [2]. Clinically, low back pain may be induced by multiple factors. However, among patients with low back pain, outpatients and inpatients with LDH account for 10~15% and 25~40%, respectively [3]. There is a high incidence of LDH but a low rate of hospitalization. Most patients with LDH can be relieved after formal conservative treatment [4].

Low back pain and sciatica are the presenting symptoms of LDH. To some extent, it can be considered that measures with the capability to improve low back pain or sciatica have the potential to treat LDH [1]. In relative to the oral and surgical treatment of low back pain in LDH, acupuncture exhibits possible advantages in avoiding possible gastrointestinal adverse reactions of oral drugs and the secondary trauma of surgery. Meanwhile, existing systematic reviews [5, 6], meta analysis [7], and randomized controlled studies [8, 9] support that acupuncture has a good analgesic effect on low back pain. The Noninvasive Treatments for Acute, Subacute, and Chronic Low Back Pain: A Clinical Practice Guideline From the American College of Physicians [10] and the Chinese Expert Consensus on “Rehabilitation Treatment of Lumbar Disc Herniation” issued by the Skeletal Muscle Professional Committee of the Rehabilitation Physician Branch of the Chinese Medical Doctor Association [1] all recommend acupuncture as a therapeutic measure for low back pain. Therefore, acupuncture was selected as the control in this study.

China, India, Brazil, and other countries have rich traditional medicine resources [11, 12], but due to the lack of timely excavation and collation, many effective folk herbal medicines or technologies are on the verge of extinction. In recent years, China and India have taken the establishment of a defensive knowledge database of traditional medicine [13, 14] and the active release of protection lists [15] as the main means and methods to effectively protect and rationally develop folk medicine, which has promoted the sustainable development of folk medicine. Folk characteristic diagnosis and treatment technology is an important part of folk medicine, Ma’s bamboo-based medicinal moxibustion therapy is a characteristic folk diagnosis and treatment technology in China, belonging to the category of herb-partitioned-moxibustion. It has been applied for over 100 years and has accumulated rich clinical experience. Among them, it exhibits the most significant effect on treating low back pain in LDH, cold due to body deficiency and conditioning of chronic fatigue syndrome.

In our previous research, 14 LDH patients with low back pain were treated with Ma’s bamboo-based medicinal moxibustion. The clinical pre-trial found that 2 cases were clinically cured, 4 cases were markedly effective and 6 cases were improved. Besides, the post-treatment VAS score, ODI score, and M-JOA score were significantly lower than those before treatment (P<0.05). It suggests that this therapy has a certain application value clinically, which, however, lacks evidence-based evaluation. There is an absence of high-level clinical efficacy data, which restricts the real participation of the therapy in clinical decision-making and, hence, hinders its clinical popularization and application to a great extent.

Folk medicine resources are not only the medical characteristics of a country, but also the embodiment of a country’s cultural and economic characteristics. They are the unique areas of independent innovation of a country and a nation’s medicine. It is essential to actively explore and sort out folk medicine resources to protect their own and national medicine culture.

Thus, we designed this multi-center, randomized controlled trials to evaluate the clinical efficacy and safety of Ma’s bamboo-based medicinal moxibustion therapy in the treatment of LDH low back pain, in order to obtain evidence-based evidence that can be replicated and has high evidence level and to provide clinicians and patients with an appropriate technology that has evidence-based support for clinical efficacy and safety. At the same time, this study will be connected with China’s national intellectual property protection platform, and provide a reference for the establishment of an effective system of mining, sorting, screening, and evaluation of folk traditional Chinese medicine characteristic diagnosis and treatment technology at the national level, so as to promote the sustainable development and popularization and application of folk traditional Chinese medicine characteristic technology, constantly enrich the diagnosis and treatment technology and means of traditional Chinese medicine promote the development of the pharmaceutical industry and benefit the people.

### Study objectives

The objective of this randomized controlled trial is to evaluate the efficacy and safety of Ma’s bamboo-based medicinal moxibustion therapy in the treatment of patients with LDH low back pain. The specific objectives are (1) to evaluate the effectiveness of Ma’s bamboo-based medicinal moxibustion therapy in improving pain, lumbar dysfunction, daily living ability, and regulating of serum β-endorphin (β-EP) and serum substance P (SP) content in patients with LDH low back pain and (2) to evaluate the safety of this therapy by recording blood routine, liver and kidney function, electrocardiogram results, and the number, nature, and severity of adverse events.

## Methods

### Study design and setting

**Fig. 1:**
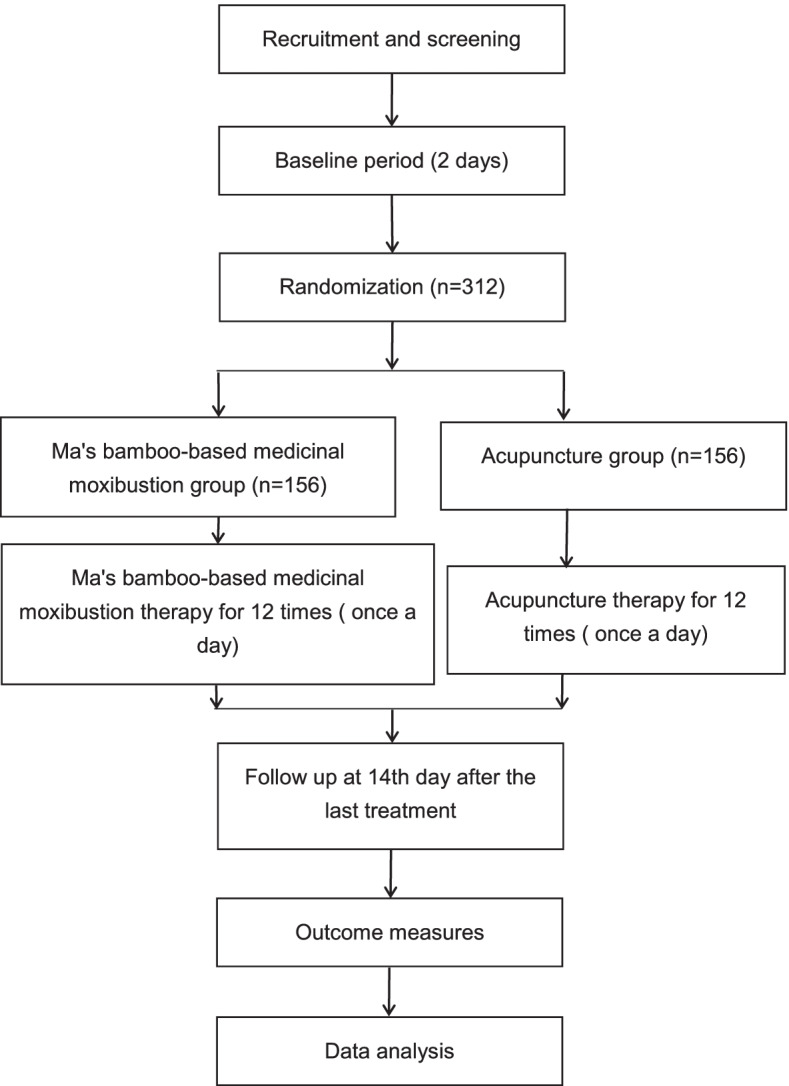
Flow chart of the trial

**Fig. 2:**
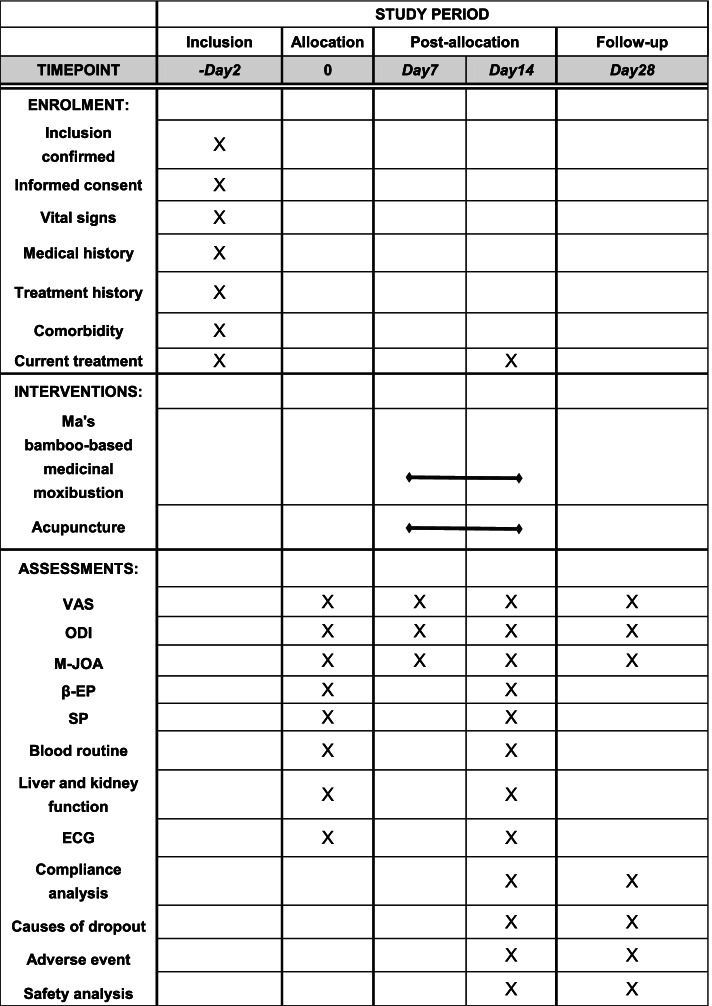
The SPIRIT figure. The schedule of enrollment, interventions, and assessments. VAS, visual analog scale; S-EP, β-endorphin; SP, substance P; ODI, Oswestry disability index; M-JOA, modified Japanese Orthopaedic Association low back pain score; ECG, electrocardiogram

This is a parallel, non-inferiority, randomized controlled clinical trial aiming to compare the effect of Ma’s bamboo-based medicinal moxibustion therapy and regular acupuncture therapy on low back pain in LDH patients. The patients will be enrolled among outpatients and inpatients from the acupuncture departments of No.1 Affiliated Hospital of Guizhou University of Traditional Chinese Medicine, No.2 Affiliated Hospital of Guizhou University of Traditional Chinese medicine. Eligible patients will be randomly assigned to a Ma’s bamboo-based medicinal moxibustion therapy group and an acupuncture group in a 1:1 ratio. All items from the World Health Organization Trial Registration Data Set are shown in Table 1. The study flowchart is provided in Fig. 1. A Standard Protocol Items: Recommendations for Interventional Trials (SPIRIT) checklist and figure are provided respectively in Additional file 1 and Fig. 2.

**Table 1:** All items from the World Health Organization Trial Registration Data Set (SPIRIT checklist, item 2b)

Data category	Information
Primary registry and trial identifying number	http://www.chictr.org.cn; ChiCTR2000038725
Date of registration in primary registry	29 September 2020
Secondary identifying numbers	-
Source(s) of monetary or material support	National Key Research and Development Project of China (2019YFC1708403)
Primary sponsor	Institute of Chinese Medical History and Literature, China Academy of Chinese Medical Sciences
Secondary sponsor(s)	-
Contact for public queries	Jing Fu; 1040684089@qq.com
Contact for scientific queries	Jing Fu, Guizhou University of Traditional Chinese Medicine, Guiyang 550002, China.
Public title	Folk diagnosis and treatment technology in lumbar disc herniation study
Scientific title	Ma’s bamboo-based medicinal moxibustion therapy of low back pain in lumbar disc herniation: study protocol for a randomized controlled trial
Countries of recruitment	China
Health condition(s) or problem(s) studied	Lumbar disc herniation, low back pain
Intervention(s)	Ma’s bamboo-based medicinal moxibustion group
Key inclusion and exclusion criteria	***Inclusion criteria*** Patients who meet all of the following criteria will be enrolled in the trial: (1) patients who meet the above diagnostic criteria and pathological classification, with the lesion located in L3–S1; (2) patients aged 18–65 years, males or females; (3) patients with the course of disease ≥6 months; (4) drug users with drug withdrawal for 4 weeks, and non-drug users with discontinued treatment for 2 weeks; (5) patients with 3 ≤VAS scores ≤6; (6) willingness to sign written informed consent. ***Exclusion criteria*** Patients who meet any of the following criteria will be excluded from the trial: (1) patients with acute onset of low back pain in lumbar disc herniation; (2) patients with low back pain caused by other causes; (3) patients with other diseases requiring combined treatment using anti-inflammatory and analgesic drugs; (4) patients during pregnancy,lactation or pregnancy preparation; (5) patients with rash, skin damage, ulcers or other infectious diseases at the waist; (6) patients with serious diseases of the heart, liver, kidney,blood system, etc.; (7) patients with mental disorders or communication disorders who could not cooperate; (8) patients with high fever and yin deficiency.
Study type	Multicentre prospective randomized trial
Date of first enrolment	11 November 2020
Target sample size	312
Recruitment status	Recruiting
Primary outcome(s)	Visual analog scale (VAS)
Key secondary outcomes	Oswestry disability index (ODI); modified Japanese Orthopaedic low back pain (M-JOA) score; serum β-endorphin (β-EP); serum substance P (SP)

The protocol has been approved by the ethical committee of No. 1 Affiliated Hospital of Guizhou University of Traditional Chinese Medicine (ethical approval number: k2020–039), and the study is registered at the Chinese Clinical Trial Registry, ChiCTR (registration number: ChiCTR2000038725; registration date: September 29, 2020).

### Patient and public participation

Participants and the public were not involved in the design or recruitment of this clinical trial. We will invite patients to be involved in the development of dissemination strategies.

### Eligibility criteria

#### Diagnostic criteria

The diagnostic criteria [2] are:

1. Lower limb radiative pain, of which the pain location was consistent with the corresponding affected innervation area
2. Lower limb sensory abnormalities, with decreased superficial sensation of the skin in the corresponding affected innervated area
3. Positive result of Lasegue test, Bragard test, Lasegue test of the unaffected side, or femoral nerve stretching test
4. Weaker tendon reflex than that of the unaffected side
5. Muscle weakness
6. Intervertebral disc herniation indicated by lumbar MRI or CT, of which the nerve compression was consistent with the symptoms, signs of the affected nerves
7. In the first five criteria, patients could be diagnosed as LDH when meeting three of them combined with item 6

#### Pathological classification [16]

1. Intervertebral disc bulging, with an outward bulge of the annulus fibrosus of the whole intervertebral disc uniformly
2. Localized herniation of the intervertebral disc, rupture of the inner layer of annulus fibrosus of the intervertebral disc, and partial herniation of nucleus pulposus
3. Intervertebral disc herniation, rupture of most annulus fibrosus of the intervertebral disc, with intact outer annulus fibrosus merely, and the nucleus pulposus confined to the intervertebral disc
4. Prolapse of the intervertebral disc, rupture of all annulus fibrosus of the intervertebral disc, nucleus pulposus tissue protruding out of the intervertebral disc and bound by the posterior longitudinal ligament
5. Sequestered disc herniation, nucleus pulposus tissue protruding out of the annulus fibrosus and posterior longitudinal ligament, and sequestered in the spinal canal

Note: In this trial, patients with a pathological classification of intervertebral disc bulging, localized herniation of the intervertebral disc, and intervertebral disc herniation will be selected for experiment according to the long-term treatment experience and practice by using Ma’s bamboo-based medicinal moxibustion therapy.

#### Syndrome classification [17]

1. Low back pain due to cold-dampness evil

The presence of a history of suffering from cold in the waist, aggravation of symptoms when the weather changes or when there is cloudy, rainy, or cold weather, with severe cold and pain in the waist, accompanied by numbness or contracture that restricts the body movements of bending or raising up, even with pain involving the lower limbs

2. Low back pain due to dampness-heat evil

Severe and heat pain in the waist, aggravation of symptoms in summer-heat and damp, cloudy and rainy weather, and possible alleviation after activities, accompanied by heavy body and feeling of sleepy, short voiding of reddish urine, yellow and greasy fur on the tongue, as well as soft and frequent pulse or wire and frequent pulse

3. Low back pain due to static blood

The presence of a history of strain or old injury in the waist, aggravation of symptoms when getting up in the morning, tired and sitting for a long while, with stiffness of the muscles on both sides of the waist, pain like the stabbing of a needle, and pain in a fixed site

4. Low back pain due to kidney deficiency

Major manifestations of soreness and weakness, with dull pain, alleviation when pressing and lifting, accompanied by weakness in legs and knees with relief when lying down and repeated onset, with fatigue and hypersomnia, and thready pulse as well

Note: In our study, patients with all the types based on the syndrome classification mentioned above will be included for experiment according to the long-term treatment experience and practice by using Ma’s bamboo-based medicinal moxibustion therapy.

#### Inclusion criteria

Patients who meet all of the following criteria will be enrolled in the trial:

1. Patients who meet the above diagnostic criteria and pathological classification, with the lesion located in L3–S1
2. Patients aged 18–65 years, males or females
3. Patients with the course of disease ≥6 months
4. Drug users with drug withdrawal for 4 weeks, and non-drug users with discontinued treatment for 2 weeks
5. Patients with 3 ≤ VAS scores ≤6
6. Willingness to sign written informed consent

#### Exclusion criteria

Patients who meet any of the following criteria will be excluded from the trial:

1. Patients with acute onset of low back pain in lumbar disc herniation
2. Patients with low back pain caused by other causes
3. Patients with other diseases requiring combined treatment using anti-inflammatory and analgesic drugs
4. Patients during pregnancy, lactation, or pregnancy preparation
5. Patients with rash, skin damage, ulcers, or other infectious diseases at the waist
6. Patients with serious diseases of the heart, liver, kidney, blood system, etc.
7. Patients with mental disorders or communication disorders who could not cooperate
8. Patients with high fever and Yin deficiency

#### Drop out criteria

Patients who meet any of the following criteria will be withdrawn:

1. Occurrence of severe adverse events
2. Other treatments received that could affect the outcome
3. Voluntary withdrawal for any reason

### Sample size calculation

The PASS software version 15.0 (NCSS, LLC. Kaysville, UT, USA.) was used to estimate the sample size. The trial is designed to determine the efficacy of Ma’s bamboo-based medicinal moxibustion therapy on low back pain in LDH and prove that its clinical efficacy is not inferior to normal acupuncture therapy. Therefore, we chose a non-inferiority trial design. According to the previous literature [8, 18] and the joint consideration of clinicians and statisticians, it is assumed that the standard deviation of VAS score reduction of patients using bamboo-based medicinal moxibustion is 2.0, the non-inferiority margin was set at 0.78, the significance level of the test is unilateral 0.05 and power of the test is 90%. According to the 1:1 grouping, the sample size required by each group was 140 cases, and the lost follow-up rate will be estimated at 10%. The final sample size of the treatment group and the control group was 156 cases respectively, and the total sample size was 312 cases.

### Randomization, allocation, and implementation procedures

Central randomization will be conducted by INTELLIGENCE FUTURE Soft Co., Ltd. (Tianjin, China). A random sequence will be generated with SAS 9.2 (SAS Institute, Inc., Cary, NC, USA) by an independent statistician who is not involved in the study, and the sequence will be stored in a central randomization system (interactive web response (IWR) system). Allocation will adopt a stratified random method. Stratified block randomization will be carried out by third party statisticians who will be not involved in any other part of the trial. The patients will be stratified based on the two different centers and randomly assigned to the Ma’s bamboo-based medicinal moxibustion group and the acupuncture group in a 1:1 ratio. The Ma’s bamboo-based medicinal moxibustion group will receive Ma’s bamboo-based medicinal moxibustion therapy. Regular acupuncture therapy will be performed on the acupuncture group. There are two investigators at each center (referred to as A and B). Investigator A will log in to the central randomization system on the computer and be responsible for inputting baseline data of patients, waiting for the system to assign random numbers and grouping, but he will not know the details of grouping and will not participate in treatment. Investigator B is responsible for performing treatment according to the grouping. Due to the nature of the intervention, it is not feasible to conceal the assignment from Investigator B and the patient, but Investigator B will not be permitted to disclose any information about his treatment to Investigator A or the patient. At each visit, A is responsible for collecting data and entering the data in the electronic data capture (EDC) system. The therapist and the data collector are separate. Statisticians are responsible for data processing and are not aware of the grouping until the end of the trial.

### Blinding

In this trial, patients and therapists will not be blinded due to the uniqueness of the two therapies. Investigators and statisticians will be blinded. Treatment, data collection, and data analysis will be conducted by trained practitioners, data collectors, and statisticians, respectively, all blinded to one another in order to ensure the reliability of the results.

The conditions for unblinding are (1) infection or other adverse events, and these patients will be withdrawn from the trial or (2) the end of the trial and data analysis.

### Interventions

All treatments will conduct in the outpatient treatment rooms of the two centers. The therapists will not be involved in the other parts of the trial. The treatments will be performed every day, for a total of 12 treatments. All the treatments will be completed within 13 days. The assessments of the treatments will be performed after treatment, on the 7th day, the 14th day, and the 28th day. All practitioners involved in the trial are trained and registered professionals. Acupuncture was performed by primary physicians with >5 years of experience at each center. A training program to standardize all manipulations and filling of a case report form (CRF) will be provided before the trial. Investigators will inform all patients about the possible inconveniences of the trial.

### Ma’s bamboo-based medicinal moxibustion group

**Fig. 3:**
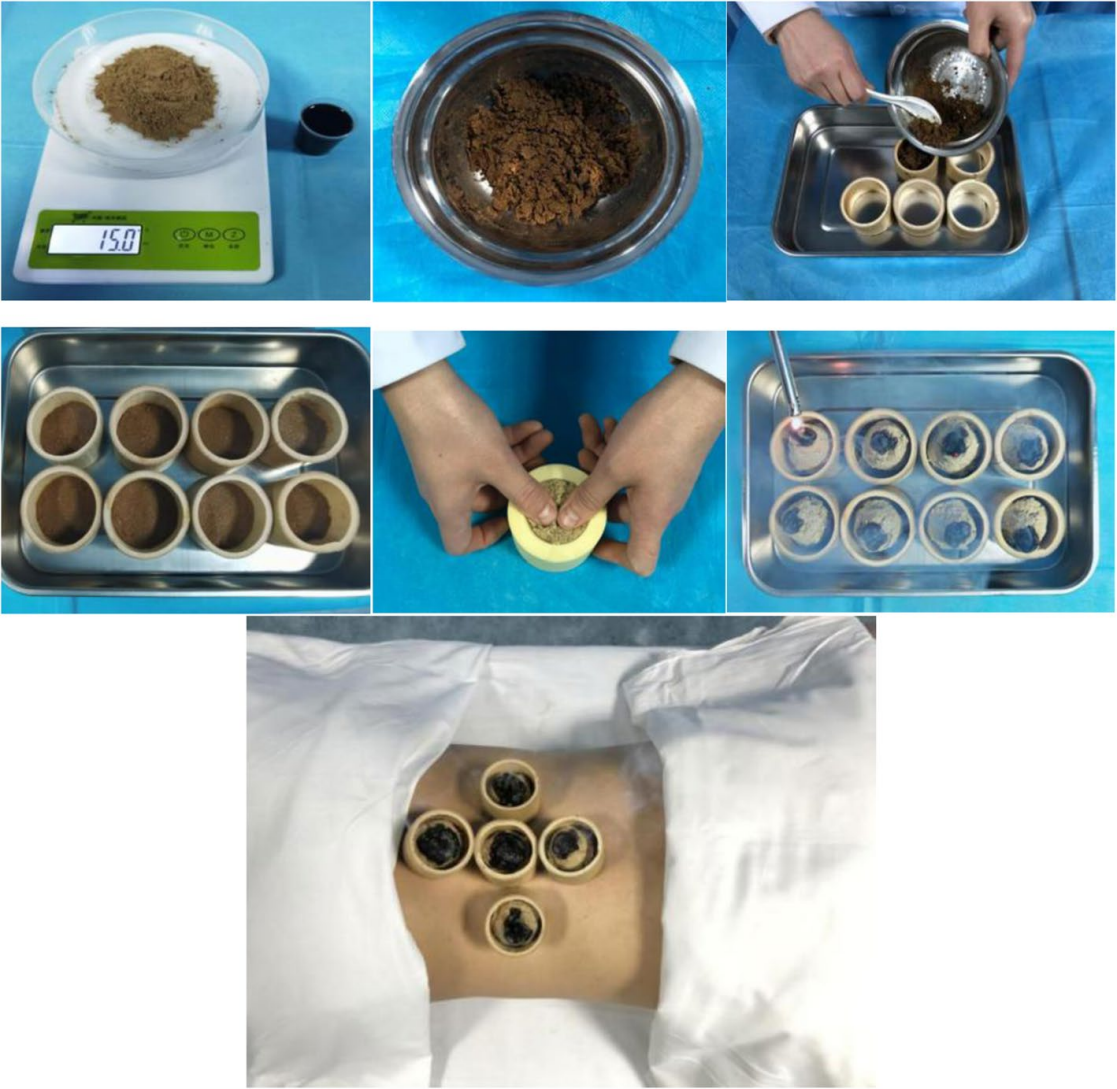
The operation process of Ma’s bamboo-based medicinal moxibustion

The specific operations of Ma’s bamboo-based medicinal moxibustion group are as follows: (1) Preparation powder of Ma’s bamboo-based medicinal moxibustion: Wei Ling Xian (*Clematidis Radix Et Rhizoma*), Du Huo ( *Angelicae Pubescentis Radix*), Rou Gui (*Cinnamomi Cortex*), Xi Xin (*Asari Radix Et Rhizoma*), Chuan Xiong (Chuanxiong Rhizoma), Sang Ji Sheng (*Taxilli Herba*), and Gui Zhi (Ci*nnamomi Ramulus*) (250g each) will be mixed and powdered through a 300-mesh sieve and stored in a dark box for later use. (2) Preparation of medicinal wine of Ma’s bamboo-based medicinal moxibustion: Wei Ling Xian (*Clematidis Radix Et Rhizoma*), Ji Xue Ten (*Spatholobi Caulis*), Chuan Niu Xi (*Cyathulae Radix*), Xi Xin (*Asari Radix Et Rhizoma*), Du Huo (*Angelicae Pubescentis Radix*), Chuan Xiong (*Chuanxiong Rhizoma*), Sang Ji Sheng (*Taxilli Herba*), and Rou Gui (*Cinnamomi Cortex*) (200g each) will be placed in 21,000 mL liquor and spirits of 50° for 7 days for subsequent use. (3) Preparation of medicinal moxibustion tool: bamboo tube with an inner diameter of 5cm is taken with the stem nodes removed, and cut with a hacksaw according to the specification of 5×4cm (inner diameter × height). Then, with the removal of the bamboo bark on the surface, the edge of the bamboo tube is polished smooth and round to prepare a unique medicinal moxibustion tool. (4) Preparation of moxa cone: 3 g of moxa (Suzhou Medical Appliance Factory, Suzhou, Jiangsu, China) are made into several moxa-cone with moderate hardness (2.5 cm in height and 2 cm in diameter) for reserve. (5) Operation: 15g of medicinal powder and 20 ml of medicinal wine are mixed evenly, and the finished product is then placed into the medicinal moxibustion tool to press into the medicinal cake. Moxa cone will be placed on the compressed powder and ignited for preheating. The patients are informed to take the prone position with the moxibustion sites exposed. L1-L5Jia ji (EX-B2), Shenshu (BL23), Mingmen Point (GV4), and Yaoyangguan (GV3) are selected for moxibustion. The acupoint positioning is performed in accordance with the nomenclature and location of acupuncture points (GB/T 12346-2006), [19]. The therapist should ensure that the moxibustion site of the patient is continuously warm without burning pain. The moxa-cone are replaced after burning out, with a total of 3 cones, and the treatment is last for about 40min. The operation process of Ma’s bamboo-based medicinal moxibustion is presented in Fig. 3.

### Acupuncture group

The selection of acupoints for the acupuncture group will follow according to the major acupuncture point prescription in *the Evidence-Based Guidelines of Clinical Practice with Acupuncture and Moxibustion Low Back Pain* [20], with positioning based on Nomenclature and Location of Acupuncture Points (GB/T 12346-2006) [19]. Each patient will be instructed to take the prone position. Disposable sterile needles of 0.30×40mm and 0.30×50mm (Suzhou Medical Appliance Co., Ltd., Suzhou, Jiangsu, China) will be used for the Shenshu (BL23), Dachangshu (BL25), Weizhong (BL40), L1-L5Jiaji (EX-B2), and Ashi points. The therapists will strictly follow the basic puncture methods and techniques in China’s acupuncture operating specifications for treatment [21, 22]. After insertion of the needle, basic lifting and twisting techniques will be performed until the needling sensation of de qi is achieved.

### Combined use of drugs

During the whole period of observation, it is suggested that the patients do not use drugs or other methods for the treatment of low back pain in LDH in addition to the experimental scheme. During the treatment period, if the patients take medication by themselves or use other methods of treatment due to aggravated symptoms and unbearable lumbar pain, they will be withdrawn from the trial. The clinicians will make the appropriate treatments according to the specific conditions of the patient until the symptoms are improved.

### Outcome measurements

#### Primary outcomes

##### Visual analog scale (VAS)

We will use the visual analog scale as the primary outcome measure [23]. VAS is one of the sensitive and reliable methods for pain measurement, which is used to evaluate the degree of low back pain. VAS scores ranged from 0 points to 10 points, which were described in detail as follows: 0: painless; less than 3 points: slight pain that can be tolerable; 4–6 points: pain to affect the sleep, which can be tolerated; 7–10 points: gradually intense pain, which is unbearable and affects appetite and sleep.

#### Secondary outcomes

Oswestry disability index (ODI) [24, 25] is one of the most widely used scoring scales for functional outcome evaluation of patients with low back pain, which can be used to evaluate lumbar dysfunction. It consists of 10 questions, including pain intensity, self-care, lifting, walking, sitting, standing, interference with sleep, sexual life, social life, and tourism. A higher score may indicate a more serious dysfunction.

Modified Japanese Orthopaedic Association low back pain (M-JOA) score [26] is a frequently used scale. It includes subjective symptoms (lumbar pain, lower limb radiative pain and numbness), objective symptoms (paravertebral tenderness, muscle tension, Lasegue test, and femoral nerve stretching test), and daily behavior ability (working ability, sleep, bending, and lifting) to evaluate lumbar conditions and activities of daily living. The score ranges from 0 points to 30 points, and a lower score may reveal a milder condition.

Serum β-EP and serum SP will be used to assess the improvement of pain in LDH. Previous clinical studies [27, 28] have reported that the reduction of pain in LDH might be correlated with the levels of serum β-EP and SP, which may be considered as one of the analgesic mechanisms of reducing low back pain in LDH. Methods of detection: At baseline and after the end of treatment, 4ml venous blood samples were collected from patients on an empty stomach in both groups, which were placed in serum separation tubes. Blood samples were then centrifuged at 1000×*g* for 20 min, the supernatant was then collected and stored in a refrigerator at − 80°C for further measurement. Enzyme-linked immunosorbent assay (ELISA) was used for determination, and the operation was carried out in strict accordance with the instructions of the kit. The test is conducted by the Central Laboratory of No.1 Affiliated Hospital of Guizhou University of Traditional Chinese Medicine.

### Concomitant care

From the time of participation in this study to the end of follow-up, it is recommended that patients do not take anti-inflammatory and analgesic drugs or any other non-drug therapy for low back pain, because this may affect the objective evaluation of the efficacy of Ma’s bamboo-based medicinal moxibustion therapy. However, if the patient needs to use other treatment methods other than the treatment plan of this study, patients can apply to the investigator to withdraw from the clinical trial, and any rights and interests of the patient will not be harmed.

### Adherence

Before the start of the trial, the investigators will fully inform the patients of the benefits and the possible inconvenience of the study. MRI of the lumbar vertebrae, laboratory examination, and twelve times of treatment will be free of charge in order to improve the adherence of the patients. The baseline data of each patient (name, gender, age, address, telephone, or contacts of immediate family members) will be recorded in detail, hereby to facilitate the supervision of patients’ adherence. The cases of drop-out, withdrawal, and discontinuation will be recorded and followed up by telephone or even followed up at patients’ homes if necessary.

### Data monitoring and management

Our study group will set up a special data monitoring committee for data management and quality control, which consists of hospitals, company, and institute.

#### Quality control system

All three-level quality monitoring system was established. Clinical research associates (CRAs) were assigned for data monitoring. Level 1 monitoring requires the CRAs in the two hospitals (No. 1 Affiliated Hospital of Guizhou University of Traditional Chinese Medicine, No. 2 Affiliated Hospital of Guizhou University of Traditional Chinese medicine) to monitor the quality of the CRFs once a week, fill out the monitoring report, and report to the principal investigator (PI) in time. Level 2 monitoring requires the CRA from INTELLIGENCE FUTURE Soft Co., Ltd to conduct quality monitoring once every six months, fill out the monitoring report, and report to the project leader of INTELLIGENCE FUTURE in time. Level 3 monitoring requires the person in charge or the technical backbone at the institute of Chinese Medical History and Literature, China Academy of Chinese Medical Sciences to monitor once or twice annually.

#### Data management platform

The data management platform was set up by INTELLIGENCE FUTURE soft Co., Ltd. It includes the IWR and EDC systems. It includes nine modules, including data entry and statistics. All data will be entered using a double-entry procedure performed by two different individuals.

### Consent and assent

The study clinician obtained written consent from the patient. All subjects included in the present clinical study had voluntarily participated, signed an informed consent form (Additional file 2), and received a copy of the informed consent form. Prior to collecting any data, the consent of the patient was sought.

### Confidentiality

All data will be confidential. The CRFs will be stored in a locked filing cabinet in the team office. The data collected by investigator A of each center will be kept confidential by the data analyst and stored in the data management platform. The data management platform access account and password will be known only to Investigator A and data analyst.

### Statistical analysis

Data analysis will be conducted in accordance with the intention-to-treat principle. Missing data will be handled using linear mixed effects models. Statisticians and chief investigators will work out an analysis plan and related forms before the database lock. The analysis will include case distribution, comparison of basic values, compliance, effectiveness, factors that influence efficacy, and safety. Subgroup analysis will be performed according to pathological classification and syndrome classification. Statistical analysis will be conducted using SAS 9.2 (SAS Institute Inc., Cary, NC, USA). The Wilcoxon rank-sum test, *t* test, or variance analysis will be performed for inter-group or intra-group comparison according to data distribution. The Wilcoxon rank-sum test will be used to compare ranked data. The chi-square test will be applied to compare enumeration data. All tests will be one-sided. A *p* value <0.05 will be considered statistically significant.

### Safety monitoring

Possible adverse events during Ma’s bamboo-based medicinal moxibustion treatment include itching, scald, redness, blister, and infection. Fainting, sticking of needle, hematoma, subcutaneous hematoma, and bleeding might occur during acupuncture treatment. Therapists and subjects are informed to actively report any adverse events during the study. Researchers will track the results of safety evaluation indexes (routine blood test, liver and kidney function, ECG) before and after treatment in a timely manner, and confirm the occurrence of adverse events. Any adverse events that occur during the trial will be recorded in a CRF by researchers and patients will be treated as soon as possible. Patients may choose to withdraw from the trial due to any adverse events. The sponsor and the data management company INTELLIGENCE FUTURE will monitor the implementation of the trial protocol and filling of CRFs.

### Protocol amendments

The eligibility criteria, results, and analyses will not be modified once the first participant has been enrolled in the study. Any significant modifications to the protocol, such as study objectives, study design, eligibility criteria, sample size, results or analysis changes, will be approved by the Research Ethics Committee and the person in charge of the project leader and submitted to the relevant ethical review body.

### Dissemination plans

Data will be provided to the investigator responsible for the study. These results will be disseminated through presentations at academic and clinical conferences and will be published in peer-reviewed journals.

## Discussion

Ma’s bamboo-based medicinal moxibustion therapy is a broad-spectrum folk therapy in china. On the basis of a modification of traditional herb-partitioned-moxibustion, it applies a unique moxibustion method and the ancestral formula for treatment, which shows an effect on treating chronic stubborn diseases such as LDH, chronic fatigue syndrome, cold due to body deficiency and other chronic stubborn diseases. In general, herb-partitioned-moxibustion can play a better role than routine moxibustion by using traditional Chinese medicinal formulae according to different individuals and diseases, which is thus widely used in clinical. It has been demonstrated that herbs-partitioned-moxibustion can alleviate low back pain in LDH [29, 30]. However, herb-partitioned moxibustion still has some disadvantages, such as small moxibustion area, weak warm stimulation of moxibustion, weak permeability, and easy to fall off to cause scald when burning the moxa-cone. Compared with herb-partitioned-moxibustion, Ma’s bamboo-based medicinal moxibustion therapy exhibits advantages in the use of the ancestral formula for treatment and the addition of a bamboo tube as a medicine moxibustion tool. Following the principle of treatment with syndrome differentiation and by using an ancestral formula, Ma’s bamboo-based medicinal moxibustion has a strong pertinence and can improve the curative effect. Besides, bamboo tube is featured by compact texture and good adsorption, which can increase the coverage area of moxibustion, facilitate the control of the dose of drugs and moxa, is simple and safe to operate, and reduce the risk of scald.

The synergistic effect of drugs, moxibustion, and acupoints constitutes the therapeutic mechanism of Ma’s bamboo-based medicinal moxibustion therapy. The pathogenesis of LDH shows association with the degenerative changes of the intervertebral disc, strain, trauma, pregnancy, etc. Low back pain is the primary clinical manifestation of LDH. In traditional Chinese medicine, the pathogenesis of the disease lies in wind-cold-dampness invasion, blood stasis, and kidney deficiency. It is the obstruction that causes the pain. Accordingly, the treatment should emphasize on promoting blood circulation, and dredging Qi and blood circulation. In terms of Ma’s bamboo-based medicinal moxibustion therapy for the treatment of low back pain in LDH, the traditional Chinese medicine prescriptions consist of Chinese herbal medicine in the folk selected by Ma’s family of traditional Chinese medicine in previous dynasties, which are used for external application and rubbing after processing, as well as the medication formula based on their medical practice experience for many years. According to the pathogenesis characteristics of “wind, cold, blood stasis and deficiency” of low back pain in LDH, the drug formula emphasizes on the use of drugs such as Wei Ling Xian (*Clematidis Radix Et Rhizoma*), Du Huo (*Angelicae Pubescentis Radix*), Sang Ji Sheng (*Taxilli Herba*), Xi Xin (*Asari Radix Et Rhizoma*), and Rou Gui (*Cinnamomi Cortex*). It is expected to exert the effects of dispelling wind and relieving pain, warming the interior and dispelling cold, activating blood circulation and removing blood stasis, and tonifying the liver and kidney. For the preparation of medicinal cake, different blending agents are selected according to different diseases in Ma’s bamboo-based medicinal moxibustion. For the treatment of low back pain in LDH, powder blending is performed with the application of the medicinal wine made of Ma’s traditional Chinese medicine prescription, which is useful for dispelling wind and removing dampness, promoting blood circulation, and relieving pain. It can hence stimulate skin tissue, expand capillaries, accelerate drug absorption and improve local blood circulation. Furthermore, on the basis of heat radiation to penetrate skin tissue through heat energy, moxibustion can play the role of anti-inflammation, analgesia, immune regulation, and blood circulation improvement [31]. By the burning of Ma’s bamboo-based medicinal moxibustion, the produced warm stimulation, infrared thermal radiation, medicinal chemical factors and physical factors can exert a therapeutic role through regulating the function of the human body. Meanwhile, the cuticle of skin at the selected acupoints is significantly thinner than that of the non-acupoints, which is characterized by reduced transdermal barrier effect and good skin permeability. It may be conducive to the percutaneous penetration and absorption of Ma’s medicinal cake. In addition, after the application of the Ma’s medicinal cake at the acupoint, a closed state in which sweat is difficult to evaporate and diffuse is formed locally. It can increase the moisture content of the cuticle, which may expand into a porous state after hydration to facilitate drug penetration [32].

Ma’s bamboo-based medicinal moxibustion therapy is a characteristic external therapy of traditional Chinese medicine in the folk. It possesses many advantages, such as a wide range of suitable diseases, and small requirements for the operating environment. Moreover, it can be applied by hospitals, families, and individuals without special care. It can be used by the patients themselves after explanation and demonstrations by doctors, without relying on doctors with professional technology, which is easy to learn and popularize. Meanwhile, it can exert the curative effect through transdermal absorption of drugs directly on acupoints, avoiding the first-pass effect of oral drugs through the liver and possible gastrointestinal adverse reactions. It may be suitable for long-term continuous treatment of chronic diseases, which can contribute to alleviating the problems of “difficulty and high cost of getting medical service” in ethnic areas. It may be conducive to improve the service capacity of folk medicine and promote the healthy development of ethnic medicine. Besides, it may benefit the improvement of the health care level of the people and health service ability in grassroots.

The trial has certain limitations. This protocol is an observational study of clinical efficacy, and most of the efficacy evaluation indexes are recorded by subjective scale. Besides, there is a lack of research based on imaging despite the detection of changes in laboratory indexes. In our subsequent research, we will continue to optimize the study design, and take into consideration of more objective indicators to conduct a more comprehensive study on the mechanism of the proposed therapy in LDH to further verify the curative effect.

## Trial status

The trial is currently recruiting patients and is expected to end in December 2021. The first patient was enrolled in November 2020.

## Supplementary Information

**Additional file 1.** SPIRIT 2013 checklist: recommended items to address in a clinical trial protocol and related documents.

**Additional file 2.** Consent form.

## Data Availability

The datasets generated during the study are available from the corresponding author upon request.

## Abbreviations

LDH: Lumbar disc herniation
VAS: Visual analog scale
S-EP: β-endorphin
SP: Substance P
ODI: Oswestry disability index
M-JOA: Modified Japanese Orthopaedic Association low back pain score
SPIRIT: Standard Protocol Items: Recommendations for Interventional Trials
EDC: Electronic data capture
IWR: Interactive web response
CRF: Case report form
PI: Principal investigator
