# Supplementary material for: Ma’s bamboo-based medicinal moxibustion therapy of low back pain in lumbar disc herniation: study protocol for a randomized controlled trial
Source: Trials. 2022 May 28;23:446. doi: 10.1186/s13063-022-06382-x (PMC9145155; doi:10.1186/s13063-022-06382-x)
Supplement: Supplementary file 2 — Additional file 2. Consent form. [file 13063_2022_6382_MOESM2_ESM.docx]

知情同意书 ·知情告知页

亲爱的患者：

医生已经确诊您为腰椎间盘突出症腰痛。我们将邀请您参加一项马氏竹技药灸疗法治疗 腰椎间盘突出症腰痛临床随机对照研究,本研究为国家重点研发计划项目，课题编号： 2019YFC1708403。本研究方案已经得到贵州中医药大学第一附属医院伦理委员会审核，同意 进行临床研究。

在您决定是否参加这项研究之前，请尽可能仔细阅读以下内容。它可以帮助您了解该项 研究以及为何要进行这项研究，研究的程序和期限，参加研究后可能给您带来的益处、风险 和不适。如果您愿意，您也可以和您的亲属、朋友一起讨论，或者请医生给予解释，帮助您 做出决定。

一、研究背景和研究目的

1.1疾病负担和治疗现况

腰椎间盘突出症( lumbar disc herniation，LDH) 是由腰椎间盘变性、纤维环破裂、髓 核组织突出压迫和刺激腰骶神经根、马尾神经所引起的一种综合征，常发生于青、中年，男 性多于女性，好发部位为L4/5、L5/S1，占 90%以上。腰痛是LDH的首发症状，疼痛一般在腰 骶部，大多为酸胀痛，可放射到臀部，反复发作，久坐、久站或劳累后加重，休息后缓解。 导致腰痛的病种繁多，但因腰痛就诊的患者中，病因为LDH的门诊患者占到了10%～15%、住院 患者占到了25%～40%LDH。腰椎间盘突出症发生率较高，多数本病患者经正规保守治疗症状可 以得到缓解。

因腰痛和坐骨神经痛为LDH的主要症状，从某种程度上可以认为，能够改善腰痛或坐骨神 经痛的措施，具有治疗腰椎间盘突出症的潜力。

目前针对腰椎间盘突出症腰痛的治疗方法包括健康教育、中医治疗、运动疗法、手术治 疗、口服药物或硬膜外注射及物理因子治疗等。相对于临床常用于治疗LDH腰痛的药物口服、 手术治疗来说，针刺避免了口服药物可能会产生的胃肠道不良反应，也避免了手术治疗对机 体产生的二次创伤，且现有的系统综述、Meta分析、随机对照研究表明针刺对于腰痛具有良 好的镇痛效果，故逐渐得到广大医患的认可。美国医师学会发布的急性、亚急性和慢性腰痛 的非介入性治疗临床实践指南、中国医师协会康复医师分会骨骼肌肉专业委员会发布的“腰 椎间盘突出症的康复治疗”中国专家共识都推荐针刺作为腰痛的治疗措施，因此本次研究选 用针刺作为对照组。

“马氏竹技药灸”是一种特殊的传统民间医技，属于隔药灸中的一种，是国家中医药管理 局行业专项“中医药传统知识保护研究”西南分中心选送的优秀项目，该方法使用已有一百 多年历史，以其独特的灸疗方法和配方对诸多疑难杂症的治疗积累了丰富的临床经验，其中 以“药灸”治疗LDH腰痛、体虚感冒及调理慢性疲劳综合症效果最为显著。

“马氏竹技药灸”与临床常用的隔物灸相比，以家传药方为主，增加了竹圈外围，便于 控制药物和艾绒的剂量，操作更加方便，在治疗LDH腰痛上疗效显著，得到患者的好评肯定， 尤其适合社区和基层医疗单位、家庭使用。课题组前期通过苗医弩药液结合温针灸治疗寒湿 型腰椎间盘突出症、针刺结合弩药灸治疗腰椎间盘突出症研究发现，将药物与灸法相结合， 往往能收到优于单用药物，或者单用灸法的治疗效果，且课题组前期通过马氏竹技药灸疗法 治疗14例腰椎间盘突出症腰痛临床预实验发现，临床痊愈2例，显效4例、好转6例，无效2例 且治疗后患者的VAS评分、ODI评分、M-JOA评分较治疗前明显降低，差异具有统计学意义。但 鉴于目前相关研究较少，尚未得到挖掘整理，缺乏科学的临床疗效依据，很大程度上阻碍了 其推广和开发。

1.2本研究目的

本研究采用平行、随机对照的方法，在以往研究的基础上，对民间医技“马氏竹技药 灸”疗法治疗腰椎间盘突出症腰痛的临床疗效及安全性进一步研究，以期对马氏竹技药灸疗 法治疗腰椎间盘突出症腰痛的临床疗效提供更加系统、客观、科学的评价。

1.3研究参加单位和预计纳入参试者例数

本试验将在贵州中医药大学第一、二附属医院同时进行，预计将有312名受试者自愿参 加。

二、哪些人不宜参加研究

（1） 腰椎间盘突出症腰痛急性发作；

（2） 腰痛由其他病因引起；

（3） 合并其他疾病且需要服用消炎镇痛药物治疗；

（4） 妊娠、哺乳期、或备孕期；

（5） 体质易过敏；

（6） 腰部有皮疹、皮肤破损、溃疡或其他感染性疾病；

（7） 存在精神障碍或沟通障碍不能配合者；

（8） 高热、阴虚患者。

注： 凡符合上述任何一条的患者，均不宜参加。

另外还有：

1） 正参加其它临床试验的患者； 2） 研究人员认为其他原因不适合临床试验者。也将不宜参 加本研究。

三、如果参加研究将需要做什么？

1．在您入选研究前，您将接受以下检查以确定您是否可以参加研究： 医生将询问、记录您的 一般情况，包括性别、年龄、身高、体重、居住环境、受教育程度、病史等，并进行相关的 体格检查以及配合研究人员提供手部照片。

您需要配合进行血常规、肝功能、肾功能、常规心电图、腰椎MRI检查。 您是合格的纳入者，您可自愿参加研究，签署知情同意书。

如您不愿参加研究，我们将按您的意愿施治。

2. 若您自愿参加研究，将按以下步骤进行：

（1） 本研究采用多中心、随机对照的方法进行研究，采用计算机软件制定的随机化方案，如 果您符合入选标准并愿意参加本研究，医生将根据计算机软件提供的随机分组结果，告知您 将得到马氏竹技药灸治疗或普通针刺治疗，您分别有1/2的可能性被分入这2个不同组中的任 何一个，您和您的医生都无法事先知道和选择治疗方法，各组治疗方法均安全、 有效。马氏竹技 药灸治疗每次取腰夹脊（双） 、肾俞（双） 、命门、腰阳关穴、每次灸3壮，施灸40分钟，每日1 次，6次为一个疗程，共治疗2个疗程，疗程之间休息1天，共计12次。针刺治疗每次取肾俞、 大 肠俞、 委中、 腰夹脊、 阿是穴， 每次留针 30 分钟， 每日1次，6次为一个疗程，共治疗2个疗程， 疗程之间休息1天，共计12次。本研究中使用的药粉及药酒为马氏传承人独家配方， 艾绒为苏州医 疗用品厂生产。针灸针为苏州医疗用品厂有限公司生产的华佗牌一次性使用无菌针灸针 ， 获得生产许可（生产企业许可证： 苏食药监生产许 2001－0020 号， 注册证号： 苏食药监械准字 2012 第 2270864） ，规格： φ0.30×40mm、 φ0.30×50mm。

（2） 治疗观察将持续4周。

（3） 你需配合于治疗前、1个疗程结束后、2个疗程结束后、2周随访时按血清β-内啡肽、血 清P物质（随访期不测定） ，视觉模拟评分（visual analogue scale，VAS） 、腰功能障碍评 分量表( Oswestry disablity indes,ODI)、改良日本骨科学会腰痛评分法( M-JOA)分别进行 含量及评分测定。

3. 需要您配合的其他事项

（1） 您必须按医生和您约定的随访时间来医院就诊及随访，您的随访非常重要，因为医生将 判断您接受的治疗是否真正起作用，同时，您还有责任向医生报告试验过程中您身体和精神 方面的任何改变，无论您认为这种改变是否与这项研究有关。

（2） 在研究期间您不能使用除本试验方案外的对腰椎间盘突出症腰痛起干预作用的药物及方 法，如您需要进行其他治疗，请事先与您的医生取得联系。

四、参加研究可能的受益

您将可能从本项研究中受益，此种受益包括您的病情有可能获得改善；本次试验期间针 对腰部进行治疗，为您提供 12 次针对腰部的免费（马氏竹技药灸/针刺）对症治疗，以及免 费手诊一次；该项目研究结果最终可能会应用、推广于临床。

尽管已经有证据提示针灸治疗腰椎间盘突出症腰痛有满意的疗效，但这并不能保证对您 肯定、有效。同时马氏竹技药灸和针刺治疗过程中，可能会出现相应的损伤及不良事件的发 生。本研究所采用的马氏竹技药灸法也不是治疗腰椎间盘突出症腰痛的唯一的方法。如马氏

竹技药灸法对您的病情无效，您可以向医生询问有可能获得的替代治疗方法。

五、参加研究可能的不良反应、风险和不适、不方便

马氏竹技药灸过程中你可能会有酸胀感、皮肤红晕，这均为药灸的正常反应。马氏竹技 药灸过程中您也可能会能出现治疗部位晕灸、皮肤瘙痒、烫伤、红疹、水泡、感染等不良反 应。当药灸时出现晕灸、皮肤瘙痒、烫伤、红疹时，您的医生会立即停止治疗，进行对症处 理； 当药灸后局部出现水泡时，只要不擦破可任其自然吸收； 若水泡过大，将用消毒针从泡 底刺破、排出水液后，再涂以龙胆紫药水； 如有继发感染，您的医生会进行相应的处理。同 时，针刺过程中您可能会有酸、麻、重、胀的感觉，这均为针刺的正常反应。针刺治疗过程 中可能会出现晕针、滞针、弯针、断针、血肿、感染等不良反应。当出现晕针时，您的医生 会立即出针，予温服糖水； 当发生滞针时，您的医生将会嘱咐您恢复原来体位，或予以循 按，缓慢出针； 当发生弯针时，您的医生根据弯针的程度、原因采用相应的处理方法，将针 缓慢起出； 当发生断针时，您的医生将会嘱咐您保持原有体位，根据针体残端的位置采用不 同的方法，将针取出； 出现血肿时，您的医生将先会予冷敷止血、再做热敷或在局部轻轻柔 按治疗。但如果针刺部位出现感染，您的医生会及时处理。

如果在研究期间您出现任何不适，或病情发生新的变化，或任何意外情况，不管是否与 研究有关，均应及时通知您的医生，他/她将对此作出判断并给与适当的医疗处理。

您在研究期间需要按时到医院随访，做一些检查，这些占用您的一些时间，也可能给您 造成麻烦或带来不方便。

六、有关费用

贵州中医药大学马氏竹技药灸研究课题组将支付您参加本项研究期间所做的与研究有关 的β-内啡肽、P物质、血常规、肝肾功能、腰椎MRI、常规心电图等检查费用，并免费提供马 氏竹技药灸和针刺治疗、以及手诊。医生将尽全力预防和治疗由于本研究可能带来的伤害。 如果在临床试验中出现不良事件，医学专家委员会将会鉴定其是否与马氏竹技药灸及针刺治 疗或试验过程有关。如发生因马氏竹技药灸及针刺治疗引起的不良反应并造成伤害，贵州中 医药大学马氏竹技药灸疗法治疗腰椎间盘突出症腰痛临床随机对照研究课题组将参照我国 《药物临床试验质量管理规范》 支付相关的治疗费用和相应的经济赔偿。

考虑到腰椎间盘突出症腰痛患者行动不便，出于人文关怀，我们将为完成试验所有项目 后的患者提供200元交通补贴，分12次发放，每次17元。

对于您同时合并的其他疾病所需的治疗和检查，将不在免费的范围之内。

七、个人信息的保密

您的医疗记录（研究病历/CRF、化验单等） 将完整地保存在您所就诊的医院。医生会将 化验和其它检查结果记录在您的病历上。研究者、伦理委员会和药品监督管理部门将被允许 查阅您的医疗记录。任何有关本项研究结果的公开报告将不会披露您的个人身份。我们将在

法律允许的范围内，尽一切努力保护您个人医疗资料的隐私。

按照医学研究伦理，除了个人隐私信息外，试验数据将可供公众查询和共享，查询和共 享将只限于基于网络的电子数据库，保证不会泄漏任何个人隐私信息。

八、怎样获得更多的信息？

您可以在任何时间提出有关本项研究的任何问题，并得到相应的解答。

如果在研究过程中有任何重要的新信息，可能影响您继续参加研究的意愿时，您的医生 将会及时通知您。

九、可以自愿选择参加研究和中途退出研究

是否参加研究完全取决于您的意愿。您可以拒绝参加此项研究，或在研究过程中的任何时 间退出本研究，这都不会影响您和医生间的关系，都不会影响对您的医疗或有其他方面利益 的损失。

出于对您的最大利益考虑，医生或研究者可能会在研究过程中随时中止您继续参加本项研 究。

如果您因为任何原因从研究中退出，您可能被询问有关您使用试验药物的情况。如果医 生认为需要，您也可能被要求进行实验室检查和体格检查。

十、现在该做什么？

是否参加本项研究由您自己（和您的家人） 决定。

在您做出参加研究的决定前，请尽可能向你的医生询问有关问题。

感谢您阅读以上材料。如果您决定参加本项研究，请告诉您的医生，他/她会为您安排一 切有关研究的事务。请您保留这份资料。

知情同意书.同意签字页

临床研究项目名称： 马氏竹技药灸疗法治疗腰椎间盘突出症腰痛临床随机对照研究 课题承担单位: 贵州中医药大学

课题协作单位： 贵州中医药大学第一附属医院、贵州中医药大学第二附属医院

课题任务书编号： 2019YFC1708403

**同意声明**

我已经阅读了上述有关本研究的介绍，而且有机会就此项研究与医生讨论并提出问 题。我提出的所有问题都得到了满意的答复。

我知道参加本研究可能产生的风险和受益。我知晓参加研究是自愿的，我确认已有充 足时间对此进行考虑，而且明白：

● 我可以随时向医生咨询更多的信息。

● 我可以随时退出本研究，而不会受到歧视或报复，医疗待遇与权益不会受到影响。

我同样清楚，如果我中途退出研究，特别是由于药物的原因使我退出研究时，我若将 我的病情变化告诉医生，完成相应的体格检查和理化检查，这将对整个研究十分有利。

如果因病情变化我需要采取任何其他的药物治疗，我会在事先征求医生的意见，或在 事后如实告诉医生。

我同意药品监督管理部门伦理委员会或申办者代表查阅我的研究资料。

我将获得一份经过签名并注明日期的知情同意书副本。

最后，我决定同意参加本项研究，并保证尽量遵从医嘱。

患者签名： ＿ ＿ ＿ ＿ 年 ＿ ＿ 月 ＿ ＿ 日

联系电话：

我确认已向患者解释了本试验的详细情况，包括其权力以及可能的受益和风险，并给其一 份签署过的知情同意书副本。

医生签名： ＿ ＿ ＿ ＿ 年 ＿ ＿ 月 ＿ ＿ 日

医生的工作电话：
